# Recurrences and Subsequent Treatments After Curative-Intent Surgery for Localised and Locally Advanced Renal Cell Carcinoma

**DOI:** 10.1245/s10434-024-16421-3

**Published:** 2024-11-19

**Authors:** Rasmus Due Petersson, Malene H. Niebuhr, Christian Fuglesang S. Jensen, Nessn H. Azawi, Frederik F. Thomsen

**Affiliations:** 1 Department of Urology, Copenhagen University Hospital, Herlev and Gentofte Hospital, Herlev, Copenhagen, Denmark; 2 Department of Urology, Zealand University Hospital, Roskilde, Denmark

**Keywords:** Renal cell carcinoma, Recurrence, Metastatic, Metastasectomy, SBRT

## Abstract

**Background:**

There is a lack of evidence concerning recurrent patterns and treatment of repeat recurrences for surgically treated renal cell carcinoma (RCC). Thus, the objective was to describe patterns of recurrences and subsequent treatments in patients with recurrent RCC.

**Patients and Methods:**

We identified 525 patients who received surgical treatment for RCC at our institution in 2010–2015. The treatment of recurrences was classified as no active treatment, treatment with the aim to achieve no evidence of disease (NED) or systemic oncological treatment (OT). Relationships were analysed using multivariable Cox regression and log-rank analysis.

**Results:**

The median follow-up was 7.8 [interquartile range (IQR 6.5–9.4)] years. Ninety-one patients experienced a first recurrence, of which 49 received NED-aimed treatment—47 of these patients had their recurrence more than 2 years after surgery. Thirty patients experienced a second recurrence with 17 patients undergoing NED-aimed treatment. Eight patients had a third recurrence with four undergoing NED-aimed treatment. The most common locations of recurrence were pulmonary, local or multiple sites—30% and 38% of patients experienced a second or third recurrence in the same location, respectively. The 3-year overall survival estimates for patients receiving NED-aimed treatment for their first recurrence were 83.1% [95% confidence interval (CI) 72.3–93.8%] and 79.3% (95% CI 58.4–100%) for patients receiving NED following a second recurrence.

**Conclusions:**

Treatments aimed at achieving NED seem to provide good oncological control and in repeat recurrences, 50% or more were managed with repeat NED-aimed treatments.

Renal cell carcinoma (RCC) represents approximately 3% of all cancers and the incidence has increased over the last 20 years.^1,2^ Surgery with curative intent is the standard treatment for localised and locally advanced renal cell carcinoma (RCC).^3^ Still, approximately 20% of patients will experience recurrence within 5 years of surgery.^4,5^

Treatment of recurrence following curative-intent surgery for RCC includes observation, surgery or radiotherapy with the objective of achieving no evidence of the disease (NED), systemic oncological treatment or palliative management depending on patient preference, performance status and the extent of the recurrence.^3,6,7^ Previous studies have suggested a survival benefit of NED-aimed treatments, compared with tyrosine kinase inhibitors and immunotherapy.^8–16^ These studies are hampered by indication bias for treatment selection and the distinction between synchronous metastasis in a metastatic RCC and recurrence from a localized RCC has not always been clearly described.^8–16^ Further, there is limited evidence concerning treatment of different recurrence patterns and prognosis in patients who undergo subsequent surgery or radiotherapy for sequential RCC recurrences, i.e. patients treated following a second, third or fourth recurrence.

The objective of this study was to describe patterns of recurrences and subsequent treatments in patients treated with curative-intent surgery for localised and locally advanced RCC. Moreover, the event of repeat recurrences and subsequent treatments were investigated in patients with recurrent RCC who underwent surgery or radiotherapy with the objective of achieving NED following recurrence.

## Patients and Methods

This is a retrospective and consecutive study including consecutive patients who underwent surgery for non-metastatic RCC at the Department of Urology, Copenhagen University Hospital, Herlev and Gentofte Hospital in the period 2010–2015.

The study received ethical and legal approval from the Danish Society for Patient Safety according to Danish law (journal number: 2012-58-0018). Recurrences of RCC and date were prospectively registered in a departmental quality registry. Patients who had RCC in their final histopathology were identified in PatoBank (Danish national pathology registry).^17^ An electronic patient chart review was performed for all identified patients and included registration of gender, date and type of surgical procedure and final histopathology, including tumour stage, histological type, Fuhrmann grade and positive margin, and for clear cell (cc) RCC the 2003 Leibovich score was used.^18,19^ Subsequent treatments and cause and date of death were registered through patient chart review. Moreover, subsequent recurrences and treatments were registered in patients treated with surgery or radiotherapy with the objective of achieving NED. Data retrieval was performed during the fall of 2022. No patient was lost to follow-up.

Patients were followed post-operatively with systematic imaging and clinical controls according to Danish National guidelines.^20^ Recurrences were identified after either routine imaging or imaging performed due to clinical symptoms suggestive of recurrence. Recurrence was confirmed with either histological verification or unequivocal imaging. Patient treatment was determined following multidisciplinary team meetings and subsequent discussion between the patient and the treating physicians.

The treatment of recurrences was classified as no active treatment, treatment with the aim of achieving NED, i.e. metastasectomy, microwave ablation (MW) or stereotactic radiotherapy (SRBT), or systemic oncological treatment. Patients were stratified according to histological type (cc versus non-cc) and patients with ccRCC were further stratified according to the 2003 Leibovich score: low-risk, 0–2 points; intermediate-risk, 3–5 points; and high-risk, 6–11 points.

Descriptive statistics were used. Median follow-up was calculated with the reverse Kaplan–Meier method.^21^ Recurrence-free and overall survival were estimated with Kaplan–Meier curves and the log-rank test was used to test differences in recurrence between different treatment modalities following recurrence (no active treatment, systemic treatment and treatment with the objective of achieving NED). Hazard ratios (HR) for the risk of death for the different treatment modalities were estimated with Cox regression—both univariate and multivariable (adjusting for age, type of primary surgery and Leibovich score). Cumulative incidence of recurrence was calculated with competing-risk analyses where death from non-RCC causes was treated as competing events. Recurrence-free survival was calculated from the time of curative-intent surgery, while survival estimates following recurrence were calculated from the time of the recurrence of interest. All tests were two-sided, and the significance level was set to *p* < 0.05. Statistical analysis was performed with R version 4.2.1 (R Foundation for Statistical Computing, Vienna, Austria).

## Results

We identified 525 patients who underwent surgery with curative intent for non-metastatic RCC with a median follow-up of 7.8 (IQR 6.5-9.4) years (Table 1). The median age was 66 (IQR 57-72) years, 64% had pT1, 6% had positive margins and 76% had ccRCC.

**Table 1:** Baseline characteristics for all patients who were treated with surgery for non-metastatic renal cell carcinoma in 2010–2015 and for the subgroup of patients who experienced recurrence

	All	Recurrence
	*n* = 525	*n* = 91
	Median (IQR)	Median (IQR)
Age, years	66 (57–72)	63 (56–69)
	*n* (%)	*n* (%)
Gender		
Female	159 (30%)	18 (20%)
Male	366 (70%)	73 (80%)
Treatment year		
2010	70 (13%)	8 (9%)
2011	87 (17%)	17 (19%)
2012	69 (13%)	17 (19%)
2013	88 (17%)	13 (14%)
2014	106 (20%)	14 (15%)
2015	105 (20%)	22 (24%)
Surgery		
Nephrectomy	259 (49%)	70 (77%)
Partial nephrectomy	266 (51%)	21 (23%)
Positive margins		
Yes	29 (6%)	6 (7%)
No	479 (91%)	84 (92%)
Undeterminable	17 (3%)	1 (1%)
T stage		
1		
1a	227 (43%)	12 (13%)
1b	112 (21%)	15 (16%)
2		
2a	68 (13%)	17 (19%)
2b	24 (5%)	3 (3%)
2 no subgroup	8 (2%)	8 (9%)
3		
3a	65 (12%)	25 (27%)
3b	15 (3%)	5 (5%)
3 no subgroup	4 (0.5%)	4 (4%)
4	2 (0.4%)	2 (2%)
Histology		
Clear cell	400 (76%)	84 (92%)
Papillary type 1	54 (10%)	2 (2%)
Papillary type 2	28 (6%)	5 (5%)
Chromophobe	34 (6%)	–
Collecting duct	2 (0.4%)	–
Unclassified	7 (1%)	–

*IQR* inter quartile range

**Fig. 1:**
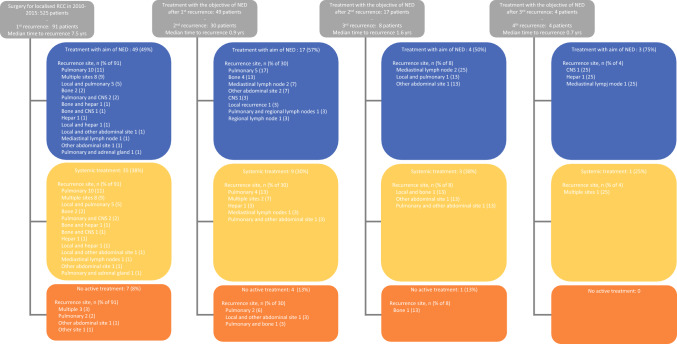
Overview of recurrences and repeat recurrences, stratified on location and subsequent treatment

Recurrence locations, stratified by subsequent treatment, are depicted in Fig. 1. In total, 91 patients experienced a first recurrence, of which 49 were eligible for NED-aimed treatment: 45 patients underwent metastasectomy (two combined with SBRT), two patients underwent SBRT and two patients underwent MW. Thirty patients experienced a second recurrence, with 17 eligible for NED-aimed treatment: 12 patients underwent metastasectomy, 4 patients underwent SBRT and 1 patient underwent MW. Eight patients had a third recurrence, of which four were eligible for NED-aimed treatment: all four patients underwent metastasectomy and four patients experienced a fourth recurrence, three of whom were eligible for NED-aimed treatment: one patient underwent SBRT and two patients underwent MW.

The most frequent primary sites of recurrence were pulmonary, local or involving multiple sites. There was a tendency for recurrences to manifest at the same site as previous occurrences. A total of 30% (9/30) of patients with a second recurrence had this recurrence in the same location as the initial recurrence. A total of 38% (3/8) of patients with a third recurrence had this recurrence in the same location as the second recurrence, while 0/4 patients had a fourth recurrence in the same location as the third recurrence. Notably, 45% (5/11) of patients who underwent NED-aimed treatments because of pulmonary recurrences encountered a second pulmonary recurrence.

The overall 5-year cumulative incidence of recurrence following curative-intent treatment was 15.3% (95% CI 12.2–18.3%). Stratified by histological subgroup the 5-year cumulative incidence of recurrence was 18.3% (95% CI 14.5–22.1%) in patients with ccRCC and 5.6% (95% CI 1.6–9.6%) in patients with non-ccRCC. Stratified by Leibovich score, the 5-year cumulative incidence of recurrence in patients with low-risk ccRCC was 5.5% (95% CI 2.5–8.5%), 29.1% (95% CI 21.4–36.8%) in patients with intermediate-risk ccRCC, and 53.7% (95% CI 38.4–68.9%) in patients with high-risk ccRCC (Table 2).

**Table 2:** Recurrences following surgery for localised renal cell carcinoma stratified by histology and for clear cell Leibovich score

	Clear cell^a^			Non-clear cell
	Leibovich 0–2	Leibovich 3–5	Leibovich 6–11	
	*n* = 220	*n* = 135	*n* = 41	*n* = 125
Five-year incidence of recurrence following curative intended surgery^b^	5.5 (2.5–8.5)	29.1 (21.4–36.8)	53.7 (38.4–68.9)	5.6 (1.6–9.6)
Five-year overall survival following curative intended surgery^c^	89.1 (84.9–93.2)	82.0 (75.5–88.5)	60.6 (45.5–75.6)	83.9 (77.5–90.4)
First recurrence				
	*n* (%)	*n* (%)	*n* (%)	*n* (%)
Number of recurrences	17 (6%)	45 (28%)	22 (48%)	7 (6%)
Median time to recurrence^d^ years (IQR)	2.8 (2.3–5.7)	2.6 (1.7–4.1)	0.7 (0.5–2.1)	0.9 (0.8–1.5)
Treatment first recurrence				
Treatment with the objective to achieve NED^e^	14 (82)	26 (56)	7 (32)	2 (29)
Systemic treatment	3 (18)	15 (33)	13 (59)	4 (57)
No active treatment	–	4 (9)	2 (9)	1 (14)
Three-year incidence of second recurrence following first recurrence for patients treated with the objective to achieve NED^c^	35.9 (7.6–64.1)	62.5 (43.6–81.4)	72.4 (38.0–100)	–
Five-year estimated overall survival following first recurrence for patients treated with the objective to achieve NED^c^	77.2 (53.8–100)	53.9 (37.5–70.4)	39.2 (18.3–60.0)	17.9 (0–49.3)
Second recurrence				
Patients treated with the objective to achieve NED after first recurrence	*n* = 14	*n* = 26	*n* = 7	*n* = 2
	*n* (%)	*n* (%)	*n* (%)	*n* (%)
Number of recurrences	5 (38%)	18 (81%)	5 (63%)	2 (100)
Median time to second recurrence,^f^ years (IQR)	1.8 (1.2–2.3)	0.7 (0.4–1.4)	2.3 (0.7–2.4)	1.6 (–)
Treatment second recurrence				
Treatment with the objective to achieve NED^e^	5 (100)	7 (39)	3 (60)	2 (100)
Systemic treatment	–	8 (44)	1 (20)	–
No active treatment	–	3 (17)	1 (20)	–
Five-year estimated overall survival following second recurrence for patients treated with the objective to achieve NED^c^	85.1 (66.0–100)	70.8 (50.8–90.7)	42.9 (0–86.8)	–

*NED* no evidence of disease

^a^A Leibovich score could not be calculated in four patients with ccRCC

^b^Cumulative incidence with 95% confidence intervals calculated with competing risk analyses where death was treated as a competing event

^c^Kaplan–Meier estimated survival probability with 95% confidence intervals

^d^Calculated from date curative intended surgery

^e^Surgery, stereotactic radiotherapy or microwave ablation

^f^Calculated from treatment of first recurrence

The median time to first recurrence from curative-intent surgery was 2 years (IQR 0.9–3.8 years), with 26 patients experiencing recurrence within 1 year, 18 between 1 and 2 years and 47 after 2 years. A total of 49 (56%) patients with a first recurrence underwent treatment with the objective of achieving NED—two patients had recurrence within the 1 year from curative-intent surgery while the other 47 patients experienced their recurrence more than 2years from surgery. A total of 49 (56%) patients with a first recurrence underwent treatment with the objective of achieving NED. Of these, 30 patients experienced a second recurrence with a median time from first to second recurrence of 0.9 years. Patients with a Leibovich score of 0–2 and 6–11 had the longest response to NED-aimed treatments with a median of 1.8 years and 2.3 years to second recurrence, respectively, while patients with a Leibovich score of 3–5 had a median time to recurrence of 0.7 years. Patients with a Leibovich score of 0–2 who underwent NED-aimed treatments after their first recurrence had the best 5-year overall survival, compared with patients with ccRCC and higher Leibovich score or patients with non-ccRCC (Table 2).

Of the 17 patients who underwent NED-aimed treatments following their second recurrence, eight experienced a third recurrence with the median time from second to third recurrence of 1.6 years. Finally, all four patients who underwent NED-aimed treatments following their third recurrence experienced a fourth recurrence with the median time from third to fourth recurrence of 0.7 years.

**Fig. 2:**
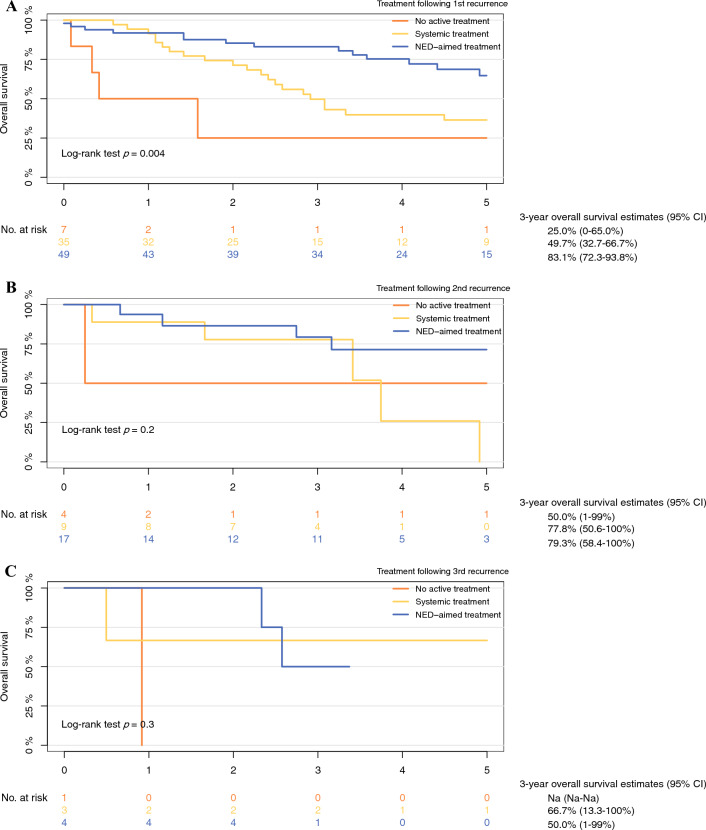
Kaplan–Meier estimates of overall survival, stratified on treatment

Patients who received NED-aimed treatments after their initial recurrence had better overall survival from the time of recurrence compared with those managed with systemic or no active treatment (log-rank test, *p* = 0.004) (Fig. 2). In univariate Cox regression analyses, patients treated with systemic treatment or no active treatment after their first recurrence had a higher risk of death (HR 2.24 [CI 95% 1.18–4.28] and HR 5.97 [confidence interval (CI) 95% 2.16–16.6], respectively), compared with patients who received NED-aimed treatments (Table 3). In multivariable analyses patients who received systemic treatment no longer had a significant higher risk of death compared to patients who underwent NED-aimed treatments (1.86 HR [CI 95% 0.95–3.64]). There was no difference in survival between treatment strategies in patients who experienced a second or third recurrence.

**Table 3:** Cox regression analyses of survival of risk of death following first recurrence

	Univariable		Multivariable	
	HR	95% CI	HR	95% CI
Treatment				
Treatment with aim of NED	Ref.		Ref.	
Systemic treatment	2.24	1.18–4.28	1.86	0.95–3.64
No active treatment	5.97	2.16–16.6	4.63	1.62–13.3
Age at recurrence				
Ten-year increase	1.36	0.93–2.00	1.23	0.82–1.85
Primary surgery				
Radical nephrectomy	Ref		Ref.	
Partial nephrectomy	0.40	0.16–1.02	0.79	0.26–2.43
Leibovich score				
0–2	Ref.		Ref.	
3–5	2.71	0.82–8.99	1.64	0.41–6.62
6–11	4.27	1.24–14.7	2.58	0.59–11.3

*HR* hazard ratio, *CI* confidence interval, *NED* no evidence of disease

## Discussion

In this retrospective study of 525 consecutive patients treated with curative intent for localised or locally advanced RCC, over 50% of the 91 patients who experienced a recurrence were able to receive treatment with the aim of attaining NED, with almost all having their recurrence more than 2 years after surgery. Additionally, subsequent NED-aimed treatments were possible in 57%, 50% and 75% of patients experiencing a second, third or fourth recurrence, respectively. As expected, patients who underwent NED-aimed treatments after their first recurrence had better overall survival compared to those who received either systemic oncological treatment or no active treatment.

The main limitations of this study are the retrospective design and the lack of a protocol for treatment selection in cases of recurrence. The latter has likely introduced selection bias that affects survival data stratified by treatment of recurrence. Moreover, because of significant advancements in systemic oncological treatments for metastatic RCC over the past decade, patients undergoing systemic oncological therapy received different treatment agents.^22^ Meanwhile, a major strength of the study is the complete follow-up, with meticulous records of recurrences and treatment trajectories for each individual who was managed with NED-aimed treatment, and its comprehensive follow-up duration. Further, the study reflects real-world clinical practice, and its results are therefore easily applicable to other centres.

The better overall survival observed in patients treated with the aim of achieving NED following their first recurrence, in comparison with those receiving systemic treatment, has been documented by prior studies.^8–16^ However, this finding warrants cautious interpretation because of anticipated selection biases whereby patients with lower metastatic burden, higher performance status and slower progressive disease were more likely to receive treatment aimed at achieving NED.^8–11,23^ Nonetheless, it suggests that pursuing NED-aimed treatments is not inferior to systemic therapy and offers the advantage of delaying the burden of significant side effects associated with systemic therapy.^24^ In historical retrospective series, individuals considered unsuitable for NED-aimed treatments were frequently used as comparators ^8–11^. This inference finds support in studies showing that the impact of metastasectomy either wanes or vanishes following propensity score matching.^16,23^ Our findings align with these observations, as we observed no significant difference in survival between patients treated with systemic oncological therapy compared to those treated with the aim of NED in multivariate analysis.

Recent research has emphasized the advantages of repeat NED-aimed treatments for pulmonary metastases, supported by earlier data from patients treated in the 1980s, which similarly showed favourable outcomes following repeat metastasectomy.^25,26^ It is plausible that part of the observed effect of metastasectomy stems from the fact that patients selected for this treatment possess a more favourable tumour biology, leading to a more beneficial natural course, irrespective of intervention.^8,23^ The current study supports this theory as fewer than 40% of patients with a Leibovich score of 0–2 who received NED-aimed treatments experienced a second recurrence within 3 years, while a higher proportion of patients with higher Leibovich scores encountered a second recurrence. Still, nearly 40% and 30% of those classified with Leibovich scores of 3–5 and 6–11, respectively, remained recurrence-free within the same timeframe. This indicates that NED-aimed treatment should not be withheld from patients with higher Leibovich scores if NED is considered achievable.

One advantage of pursuing NED-aimed treatments is the delay of exposure to potentially adverse effects associated with systemic oncological treatments.^24^ However, NED-aimed treatments carry their own risks of complications, as demonstrated by Meyer et al., who documented complications in 46% of 1102 metastasectomy cases, with 28% experiencing major complications (defined as Clavien–Dindo III–V)^27^. Therefore, the timing of NED-aimed treatment and the likelihood of achieving NED must be carefully weighed against the risk of complications. Studies such as SURTIME and CARMENA have highlighted the greater importance of systemic oncological treatment compared with cytoreductive nephrectomy (without metastasectomy) in patients with primary metastatic RCC.^28,29^

Both the European Association of Urology (EAU) and the American Urological Association (AUA) guidelines recommend post-surgery follow-up for RCC to detect local or distant recurrences during a period when the patient might still be amenable to subsequent interventions with the aim of achieving NED.^3,6,7^ Our findings support these recommendations, as nearly 50% of recurrences in the current study were amenable to treatments with the goal of achieving NED.

## Conclusion

In recurrent of RCC more than 2 years after curative-intent surgery for RCC, treatments aimed at achieving NED seem to provide good oncological control without compromising overall survival compared to systemic oncological treatments. Further, in repeat recurrences, 50% or more were managed with additional NED-aimed treatments, and around one-third of the repeat recurrences manifested in the same organ as the previous recurrence.

## Data Availability

These data have not previously been presented, and the manuscript has been submitted solely to *Annals of Surgical Oncology* and is not under consideration elsewhere.
